# Analogous modulation of inflammatory responses by the endocannabinoid system in periodontal ligament cells and microglia

**DOI:** 10.1186/s13005-020-00244-0

**Published:** 2020-11-16

**Authors:** Andreas Jäger, Maria Setiawan, Eva Beins, Ingo Schmidt-Wolf, Anna Konermann

**Affiliations:** 1 Department of Orthodontics, Medical Faculty, University of Bonn, Welschnonnenstr. 17, 53111 Bonn, Germany; 2 Department of Integrated Oncology, Center for Integrated Oncology (CIO), University Hospital Bonn, Venusberg-Campus 1, 53127 Bonn, Germany; 3 Institute of Molecular Psychiatry, Medical Faculty, University of Bonn, Sigmund-Freud-Str. 25, D-53127 Bonn, Germany

**Keywords:** Endocannabinoid system, Immunology, Inflammation, Microglia, Periodontal ligament cells

## Abstract

**Background:**

Periodontal ligament (PDL) cells initiate local immune responses, similar to microglia regulating primary host defense mechanisms in neuroinflammatory events of the central nervous system. As these two cell types manifest similarities in their immunomodulatory behavior, this study investigated the thesis that the immunological features of PDL cells might be modulated by the endocannabinoid system, as seen for microglia.

**Methods:**

A human PDL cell line and an Embryonic stem cell-derived microglia (ESdM) cell line were grown in *n* = 6 experimental groups each, incubated with cannabinoid receptor agonists arachidonoylethanolamine (AEA) (50 μM) or Palmitoylethanolamide (PEA) (50 μM) and challenged with centrifugation-induced inflammation (CII) for 6 and 10 h. Untreated samples served as controls. Quantitative real-time polymerase chain reaction was applied for gene expression analyses of inflammatory cytokines, cannabinoid receptors and ionized calcium binding adaptor molecule 1 (IBA-1). Microglia marker gene IBA-1 was additionally verified on protein level in PDL cells via immunocytochemistry. Proliferation was determined with a colorimetric assay (WST-1 based). Statistical significance was set at *p* < 0.05.

**Results:**

IBA-1 was inherently expressed in PDL cells both at the transcriptional and protein level. AEA counteracted pathological changes in cell morphology of PDL cells and microglia caused by CII, and PEA contrarily enhanced them. On transcriptional level, AEA significantly downregulated inflammation in CII specimens more than 100-fold, while PEA accessorily upregulated them. CII reduced cell proliferation in a time-dependent manner, synergistically reinforced by PEA decreasing cell numbers to 0.05-fold in PDL cells and 0.025-fold in microglia compared to controls.

**Conclusion:**

PDL cells and microglia exhibit similar features in CII with host-protective effects for AEA through dampening inflammation and preserving cellular integrity. In both cell types, PEA exacerbated proinflammatory effects. Thus, the endocannabinoid system might be a promising target in the regulation of periodontal host response.

## Background

Besides maintaining tissue integrity and homeostasis in the periodontium, periodontal ligament (PDL) cells also play an important role in regulating local immune responses [1]. In inflammatory settings, inter alia engendered by mechanical overload due to orthodontic tooth movement, activated PDL cells synthesize and secrete pro- or anti-inflammatory cytokines for the onset of immunological processes [1]. Recently, evidence was provided these resident cells furthermore have the capacity for phagocytosis, for synthesis of MHC class II molecules and for interaction with innate and adaptive immune cells upon cell-cell contact [1–3]. Even though inflammation, regulated by cytokines such as Interleukin (IL-)1ß, IL-6 and Tumor necrosis factor (TNF) α, categorically represents a protective mechanism resolving harmful and destructive stressors, persistent and excessive inflammation can get beyond physiological control [4, 5]. Consequently, chronic inflammation has to be restrained by protective pathways maintaining cellular homeostasis and balancing both the initiation and the resolution of inflammation [6]. Owing the capacity to modulate local immune responses, PDL cells can guide immunological processes towards exacerbation versus tolerance and actively impact host defense mechanisms [1].

Along with other factors arisen to have pivotal function in oral immunology, as of late the endocannabinoid system is discussed to play a role in periodontal inflammation [7]. Our previous investigations identified co-expression of cannabinoid receptors CB1 and CB2 on PDL cells, as it was seen for peripheral immune cells as well, potentially qualifying them as an important target for cannabinoid-driven regulation of periodontal immunology [8]. In addition, it was found that cannabinoids are able to promote periodontal cell adhesion and migration and thus induce cellular wound healing and regeneration processes [9]. Furthermore, CB receptor activation can facilitate osteogenic differentiation of PDL cells by upregulation of corresponding gene expression patterns and induction of mineralization processes, and presumably also in an inflammatory setting [10, 11]. This research focuses on receptor-binding endocannabinoids N-arachidonoylethanolamine (AEA) and Palmitoylethanolamide (PEA) as promising inflammatory modulators, as they are supposed to regulate cytokine networks among different cells and adjust innate and adaptive immune responses [12–14]. Here, literature predominantly attributes a protective and anti-inflammatory function to these two endocannabinoids in investigated pathologies [15–17]. AEA has already been detected in periodontal tissues and in the gingival crevicular fluid of patients with periodontitis, even though its precise role remains as much as the understanding of endocannabinoid-driven immune modulation still needs to be elucidated [18].

Analogous to the immunomodulatory features seen for resident PDL cells in the periodontium, microglia exhibit similar characteristics in the central nervous system (CNS). There, they regulate the primary events of neuroinflammatory responses and influence host defense mechanisms as much as tissue repair [19]. Upon pathological stimuli, microglia rapidly transform from a resting to an activated state enabling them to proliferation, migration, cytokine release and phagocytosis [19, 20]. A key marker for activated microglia and its accompanying features is ionized calcium binding adaptor molecule 1 (IBA-1), whose expression is supposed to be restricted to this cell type, and which is involved in the dynamic remodeling of the actin cytoskeleton underlying the mobilization process [21]. The activation states of microglia involve a phenotypic polarization into a neurotoxic M1 phenotype with production of pro-inflammatory cytokines, such as TNFα and IL-1ß, and a neuroprotective M2 phenotype with expression of anti-inflammatory factors facilitating repair mechanisms [22]. At this juncture, a multitude of investigations recently put a main focus on the endocannabinoid system. Particularly AEA represents a promising target in the guidance of microglial polarization, as they express both cannabinoid receptors that could potentially modulate their immunological features [23]. As PDL cells and microglia represent two resident cell types manifesting similarities in their immunomodulatory behavior, we postulate the thesis that the immunological features of PDL cells might also be modulated by the endocannabinoid system, as it can be seen for microglia. Thus, a potential interaction between the inflammatory response of PDL cells and the endocannabinoid system might represent a promising modulatory target in periodontal immunology.

## Methods

The aim of the study was to assess the impact of the endocannabinoids AEA and PEA on the expression of inflammatory molecules in PDL cells at rest and under simulated mechanical loading, and to investigate potential correlations between PDL cells and microglia in their immunomodulatory function and performance.

The study was performed according to the ethical principles of the World Medical Association Declaration of Helsinki. Informed consent was obtained from all human donors. The study has been independently reviewed and approved by the Ethical Committee of the University of Bonn (reference number 029/08).

### Cell culture and chemicals

A human PDL cell line purchased from Lonza (Basel, Switzerland) was cultured in Dulbecco’s modified Eagle’s medium (DMEM; Gibco; Thermo Fisher Scientific, Inc., Waltham, MA, USA) supplemented with 10% heat-inactivated FCS (Gibco) and 1 μg/ml penicillin/streptomycin (Gibco). Cells were passaged using trypsin/EDTA (Gibco) after reaching confluence.

Embryonic stem cell-derived microglia (ESdM) cell line was kindly provided by Dr. A. Zimmer, Institute of Molecular Psychiatry, University Hospital of Bonn, Germany. Microglial cell isolation and culture was performed as previously desribed [24–26]. In brief, ESdM generated from C57BL/6-ATCC ES cells, were cultured in sterile-filtered N2-medium (DMEM/F12, Gibco) supplemented with N2 supplement (Invitrogen; Thermo Fisher Scientific, Inc., Waltham, MA, USA), 0.48 mM L-glutamine (Gibco) and 1 μg/ml penicillin/streptomycin (Gibco). Cells were passaged by scraping when reaching 80% confluence. Both PDL and ESdM cells were grown in *n* = 6 experimental groups each at 37 °C in a humidified 5% CO2 atmosphere. One day before experimentation, confluent cultures were provided with serum-free medium to eliminate nonspecific cell cycle effects.

To mimic the mechanical strain, cells were challenged with centrifugation-induced inflammation (CII) for 6 h and 10 h, respectively, by horizontal microplate rotor as previously described [27, 28]. This model is based on permanent application of pressure centrifugal force at a magnitude of 33.5 g/cm^2^, equaling orthodontic forces developing in vivo as evidenced in former analyses [29].

30 min before experimentation and for the whole duration of the experiment, cells were stimulated with the CB agonists AEA (50 μM; Sigma-Aldrich, St. Louis, MO, USA) or PEA (50 μM; Sigma-Aldrich) [30, 31]. Untreated uncentrifuged cells served as controls.

Three independent replicates per cell line and condition were analyzed in duplicates for a total sample size of *n* = 6 per entity–intervention pair.

### RNA extraction, quality control and cDNA synthesis

Total messenger ribonucleic acid (mRNA) was isolated and purified from cell lysates using the RNeasy Mini Kit (Qiagen, Hilden, Germany) according to the manufacturer’s protocol. Isolated mRNA was quantified spectrophotometrically (Nanodrop; Thermo-Fischer Scientific) and its purity was determined at 260/280 absorbance ratio.

mRNA was reverse-transcribed to complementary deoxyribonucleic acid (cDNA) employing the iScript Select cDNA Synthesis Kit (Bio-Rad Laboratories, Hercules, CA, USA). The 20 μl cDNA synthesis reaction oligo(dT) primer mix was prepared according to the manufacturer’s protocol. The maximum available amount of RNA from each sample was used in each synthesis reaction with a cutoff at 1 μg total RNA as the maximum applicable. Synthesis steps were the oligo(dT) primer cDNA reaction at 42 °C for 90 min and the reverse transcriptase inactivation at 85 °C for 5 min performed on an iCycler (Bio-Rad Laboratories).

### qRT-PCR

Quantitative real-time polymerase chain reaction (qRT-PCR) was operated on a StepOnePlus™ Real-Time PCR System (Thermo-Fischer Scientific) with primers for TNFa, IL-1ß, IL-6, CB1, CB2, IBA-1 and GAPDH as housekeeping gene for data normalization of target gene expression. TaqMan® assay ID information are headed in Table 1. Negative controls of nuclease-free water were included to obviate DNA contamination in the PCR mix. Melting curve analysis verified the specificity of the PCR products. Data were analyzed by the 2^(−∆∆ C(T))^ method according to Pfaffl [32].

**Table 1:** TaqMan® assay IDs for qRT-PCR

Gene	Assay ID
AIF-1 / IBA-1	Hs00610419_g1
CNR1 / CB1	Hs01038522_s1
CNR2 / CB2	Hs00361490_m1
GAPDH	Hs02786624_g1
IL-1ß	Hs01555410_m1
IL-6	Hs00174131_m1
TNFa	Hs00174128_m1

### Proliferation assay

Cells were seeded in 96-well plates at a density of 1500 cells/well, grown for 24 h, subsequently set to serum-free conditions for 24 h and then treated as described above. After a total experimentation time of 72 h, a WST-1 assay was performed for cell proliferation measurement according to the manufacturer’s instructions (Takara, Kusatsu, Japan). In brief, the color change was observed after 3 h of incubating the cells with the dye at 37 °C, 5% CO_2_. The absorbance was determined at 450 nm with 640 nm as the reference wavelength.

### Immunofluorescence staining and expression analysis of IBA-1

PDL cells and ESdM cells were seeded on 13 mm ∅ glass coverslips (Marienfeld GmbH & Co, Lauda-Königshofen, Germany) in 24-well plates (BD Biosciences, Mississauga, Canada). After exposure to the different culture conditions as described above, cells were fixed using 4% paraformaldehyde (Sigma Aldrich) for 10 min, permeabilized with 0.05% Triton X and blocked with 2% goat serum (Abcam, Cambridge, United Kingdom) in 1% phosphate buffered saline/bovine serum albumin for 1 h. Immunofluorescence staining was achieved by incubation with anti-IBA-1 (Wako Chemicals GmbH, Neuss, Germany) for 1 h, followed by 30 min incubation with secondary Alexa Fluor 488 (Life Technologies; Thermo Fisher Scientific, Inc., Waltham, MA, USA)).

Counterstaining was done using 1 μg/ml of DAPI (Sigma Aldrich) for 10 min before mounting with Aqua-Poly/Mount (Polysciences, Inc., Warrington, PA, USA). For negative controls, primary antibodies were omitted. All incubations were performed at room temperature in the dark.

The stained slides were observed with Axio Imager M2 (Carl Zeiss Jena GmbH, Jena, Germany) at 40 x magnification. Images were taken with 70 ms illumination time in the green channel and 1 ms illumination time in the DAPI channel.

The mean fluorescence intensity (MFI) for each image was determined according to the quantitative analysis of fluorescence staining using ImageJ software [33]. In brief, images were converted into 8-bit. The regions of interest (ROI) were selected randomly per cell, analyzing each cell per image. Then, mean grey values for each cell were calculated, following determination of the mean grey value for each image by calculating the ratio total mean grey value / number of cells per image.

### Statistical analysis

One-way ANOVA was applied for comparison between groups followed by Bonferroni correction. Gene induction was analyzed with one sample t-test after application of the 2^(−∆∆ C(T))^ method (GraphPad Software, San Diego, CA, USA). Results are expressed as mean ± SEM (*n* = 6). The level for statistical significance was set at *p* < 0.05 (* *p* < 0.05, ** *p* < 0.01, *** *p* < 0.001).

## Results

### AEA deregulates inflammatory cytokine expression aroused by CII in PDL cells and in microglia

**Fig. 1:**
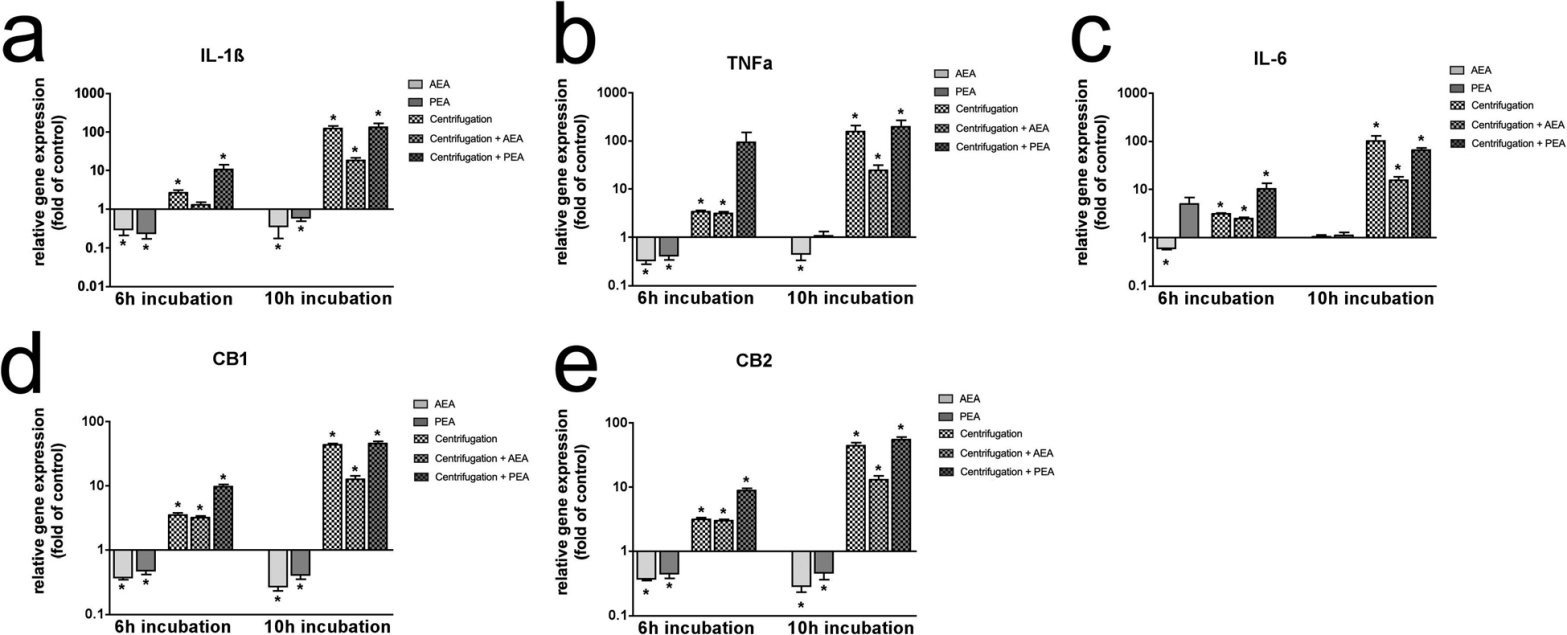
Transcriptional regulation of inflammatory cytokines and CB receptors in PDL cells upon CII and endocannabinoid treatment. Gene expression analyses were performed for IL-1ß (**a**), TNFa (**b**), IL-6 (**c**), CB1 (**d**) and CB2 (**e**) upon challenge with or without AEA (50 μM) or PEA (50 μM) under serum-free conditions in combination with or without CII of PDL cells for 6 h or 10 h, respectively. Analyses were performed via qRT-PCR. Values represent the mean ± SEM (*n* = 6) of the relative differential gene expression (fold of control). * statistically significant compared to unstimulated control (*p* < 0.05, one sample t test)

**Fig. 2:**
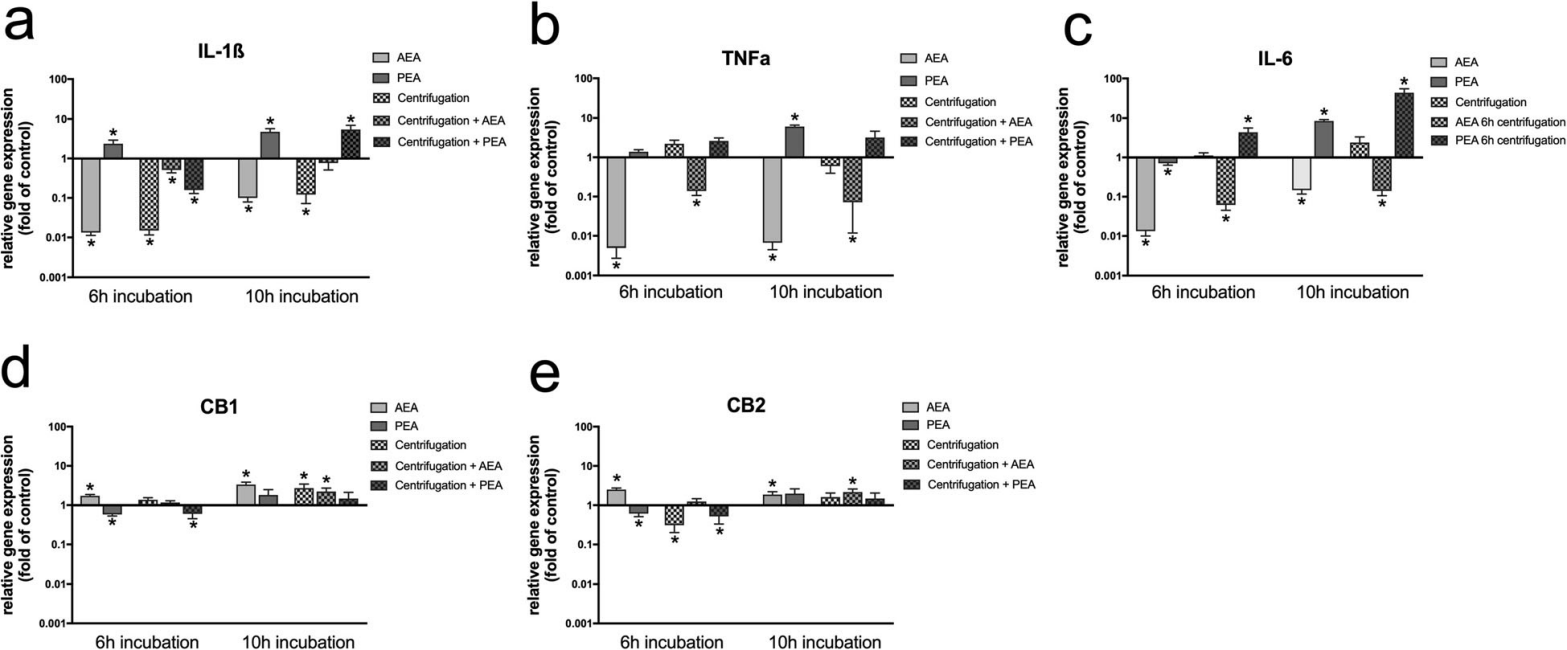
Transcriptional regulation of inflammatory cytokines and CB receptors in microglia upon CII and endocannabinoid treatment. Gene expression analyses were performed for IL-1ß (**a**), TNFa (**b**), IL-6 (**c**), CB1 (**d**) and CB2 (**e**) upon challenge with or without AEA (50 μM) or PEA (50 μM) under serum-free conditions in combination with or without CII of Embryonic stem cell-derived microglia (ESdM) cells for 6 h or 10 h, respectively. Analyses were performed via qRT-PCR. Values represent the mean ± SEM (*n* = 6) of the relative differential gene expression (fold of control). * statistically significant compared to unstimulated control (p < 0.05, one sample t test)

Analysis of transcriptional expression changes for the key inflammatory markers IL-1ß, IL-6 and TNFa as much as for CB1 and CB2 receptors was performed upon challenge with AEA or PEA combined with or without centrifugation in PDL cells (Fig. 1) and in ESdM cells (Fig. 2).

In PDL cells, AEA significantly downregulated inflammatory cytokines and CB receptors after stimulation for 6 h and 10 h under unstressed conditions.

CII upregulated inflammatory molecules in a time-dependent manner, which was also evident for CB receptors. After 10 h, IL-1ß was upregulated 124.8-fold ±19.7, TNFa 155.8-fold ±56, IL-6100.5-fold ±30.2, CB1 43.2-fold ±2.6 and CB2 44.1-fold ±4.8. Except for IL-6 after 10 h, an additional significant increase of all target genes was evident for CII plus PEA. After 10 h, values of 134.9-fold ±33.9 for IL-1ß, 195.6-fold ±74.9 for TNFa, 12.5-fold ±1.6 for CB1 and 12.9-fold ±1.9 for CB2 could be noted.

Contrarily, CII plus AEA potently inhibited transcriptional increases by CII. After 10 h, values decreased to 18.2-fold ±3.2 for IL-1ß, 24.3-fold ±7.1 for TNFa, 15.5-fold ±2.9 for IL-6, 45.3-fold ±4.1 for CB1 and 54.6-fold ±4.9 for CB2.

In ESdM cells, CII plus AEA downregulated inflammatory molecules, but interestingly even stronger under unstressed conditions both after 6 h and 10 h. Highest downregulations were seen for AEA after 6 h with 0.003-fold ±0.0 for TNFa, and 0.088-fold ±0.0 for IL-6. Only for IL-1ß, expression was higher for CII plus AEA both after 6 and 10 h compared to CII only. Challenge with PEA upregulated proinflammatory cytokines with and without CII. After 10 h PEA challenge plus CII, increases were 4.9-fold ±1.7 for IL-1ß, 3.4-fold ±2.4 for TNFa and 68.4-fold ±12.3 for IL-6.

Regarding CB receptor-regulation in ESdM cells, transcriptional increase due to CII with or without AEA or PEA could also be observed analogous to PDL cells, but to an extent with highest values of 3.0-fold ±1.2 that can be regarded as methodological variation.

### Proliferation of PDL cells and ESdM cells is similarly modulated by CII and by endocannabinoid treatment

**Fig. 3:**
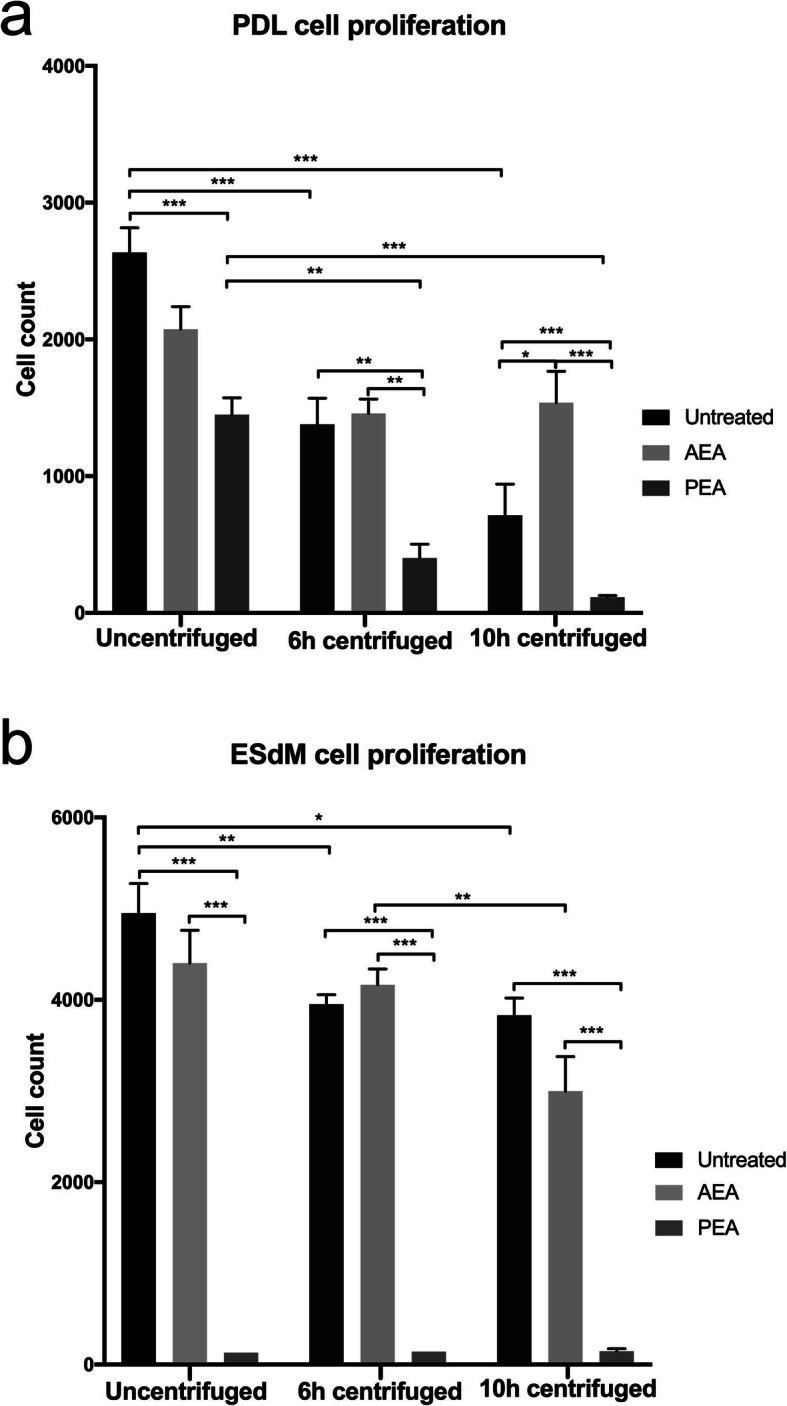
Proliferation of PDL cells and microglia subject to CB receptor agonist modulation in an unstressed versus CII environment. PDL cells (**a**) and Embryonic stem cell-derived microglia (ESdM) cells (**b**) were subjected to AEA (50 μM) or PEA (50 μM) treatment for a total incubation time of 72 h. Samples were additionally treated with or without CII for the first 6 h or 10 h of the total incubation time. Unstimulated cells served as control. Readouts were subsequently performed by a WST assay. Values represent the mean ± SEM (n = 6) of the total cell count. Statistical analyses were done with one-way ANOVA for the comparison between groups followed by Bonferroni correction. The level for statistical significance was set at *p* < 0.05 (* *p* < 0.05, ** *p* < 0.01, *** *p* < 0.001)

In both cell types, CII reduced cell proliferation in a time-dependent manner (Fig. 3). In PDL cells, proliferation decrease correlated with CII duration, with 2635 cells ±182 for uncentrifuged controls, 1379 cells ±191 after 6 h CII and 714 cells ±228 after 10 h CII. In ESdM cells, the same pattern could be observed, even though significantly less with 4951 cells ±324 for uncentrifuged controls, 3953 cells ±105 after 6 h CII and 3831 cells ±188 after 10 h CII.

Administration of AEA did not noticeably modify proliferation compared to controls in ESdM cells, neither unstressed nor upon CII. Contrarily, AEA was able to partly inhibit cell reduction by CII in PDL cells. PEA synergistically reinforced the inhibition seen for CII with stepwise reduction of PDL cell count to 115 cells ±13 after 10 h. In ESdM cells, PEA reduced the cell count to values between 130 and 147 cells regardless of whether CII was added or not.

### Microglia marker IBA-1 is inherently expressed in PDL cells on transcriptional and on protein level and exhibits signal changes due to CII and AEA

**Fig. 4:**
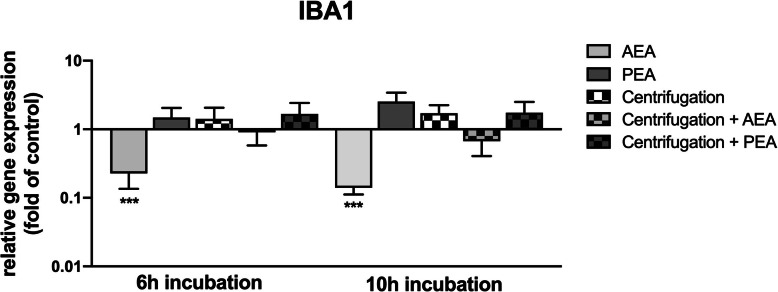
Transcriptional regulation of IBA-1 in PDL cells upon CII and endocannabinoid treatment. Gene expression analyses for IBA-1 were performed upon challenge with or without AEA (50 μM) or PEA (50 μM) under serum-free conditions in combination with or without CII of PDL cells for 6 h or 10 h, respectively. Analyses were performed via qRT-PCR. Values represent the mean ± SEM (n = 6) of the relative differential gene expression (fold of control). The level for statistical significance was set at *p* < 0.05 (* *p* < 0.05, ** *p* < 0.01, *** *p* < 0.001)

Gene expression analyses of IBA-1 revealed innate occurrence of this microglia marker molecule in PDL cells with a basal constitutive expression level in unstimulated cells of 0.49% in the mean. In stimulation experiments, challenge with AEA significantly downregulated its expression when operating alone, resulting in a decrease to 0.2-fold ±0.0 after 6 h stimulation and to 0.1-fold ±0.0 after 10 h stimulation. This effect was counteracted when CII was additionally applied, which is graphically illustrated in Fig. 4.

**Fig. 5:**
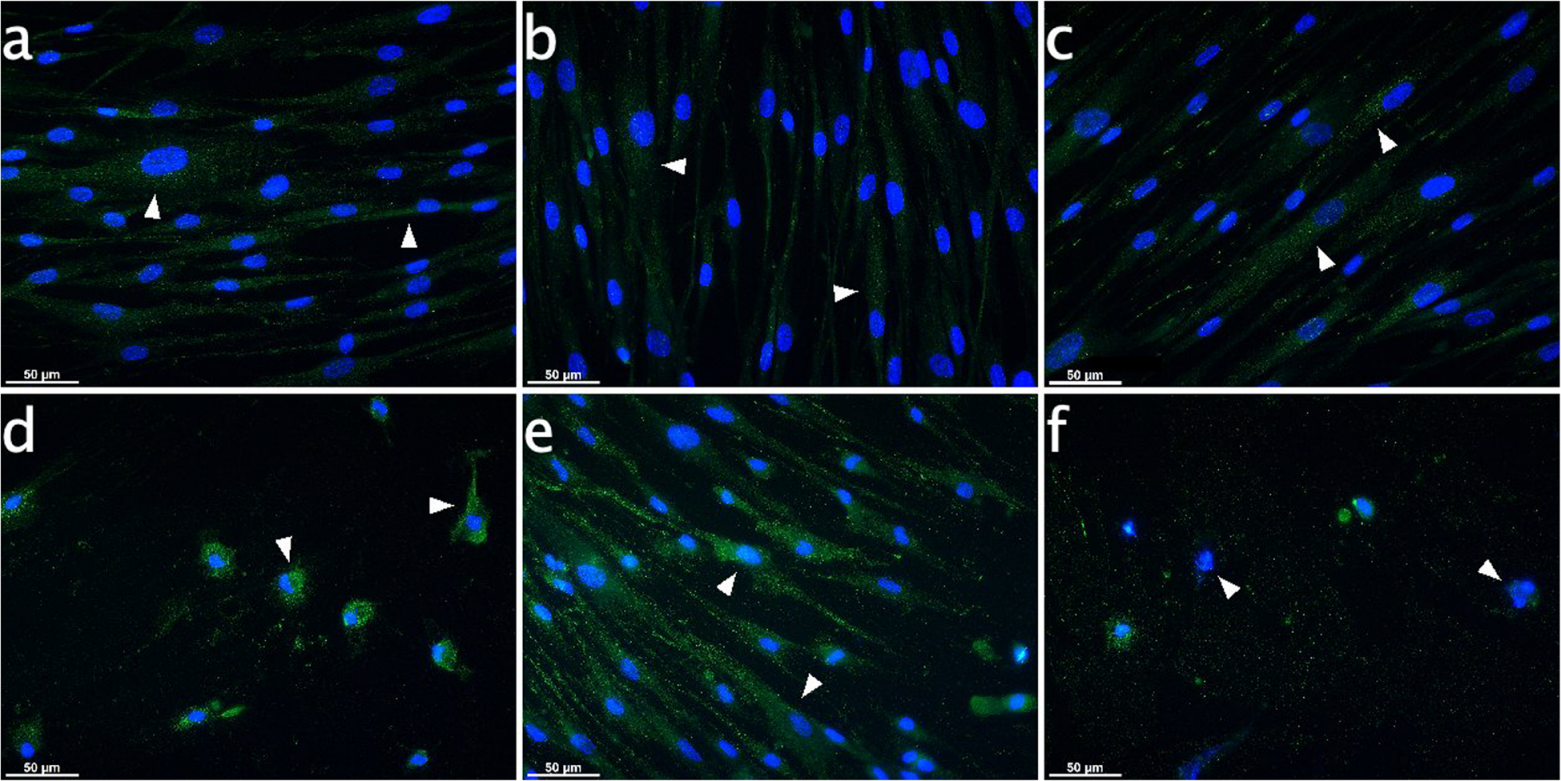
IBA-1 expression in PDL cells subject to CB receptor agonist modulation with or without CII for 6 h. Immunocytochemical analyses for IBA-1 (green) after 6 h stimulation revealed inherent expression in cultured PDL cells. The IBA-1 signals were distributed equally in the cytoplasm for controls and with or without endocannabinoid treatment (arrowheads on Fig. **a**, **b**, **c**), exhibiting dotted structures. IBA-1 MFI values increased in all groups after CII, although signal distribution concentrated around the nuclei due to changes in cell morphology and loss of cells (arrowheads on Fig. **d**, **f**). However, AEA was able to reverse effects aroused by CII, and even intensified IBA-1 signals. **a**: Control, **b**: AEA-treated (50 μM), **c**: PEA-treated (50 μM), **d**: 6 h CII, **e**: 6 h CII plus AEA-treated (50 μM), **f**: 6 h CII plus PEA-treated (50 μM). Visualization was performed with Alexa 488 (green) secondary antibody. Nuclei were stained with DAPI (blue). Scale bar is 50 μm

Immunofluorescence staining of IBA-1 in PDL cells evidenced equally distributed cytoplasmatic signal with additonal brightly stained small granules. Here, immu- nohistochemical reaction products were present uniformely, and local concentrations were not seen. These characteristics were typical of controls and after 6 h of stimulation with AEA or PEA (arrowheads in Fig. 5a-c). MFI values ranged between 34.7 ± 2.6 and 38.6 ± 1.8. MFI and number of dotted structures distinctly increased in all groups by CII (Fig. 5d-f). In the CII group and the CII plus PEA stimulated group, morphological changes of cells into roundish structures with loss of spindle-shape and thus a signal concentration around the nucleus could be observed (arrowheads in Fig. 5d&f). Furthermore, a significant cell loss was evoked by CII, and even stronger by additional PEA challenge. MFI increased to 55.5 ± 3.4 for CII and 51.5 ± 2.5 for CII plus PEA-treated cells. Conversely, AEA completely abolished CII-induced effects, as cell morphology and cell number were equal to controls. Moreover, IBA-1 signal intensity increased to 80.7 ± 2.2 (Fig. 5e).

**Fig. 6:**
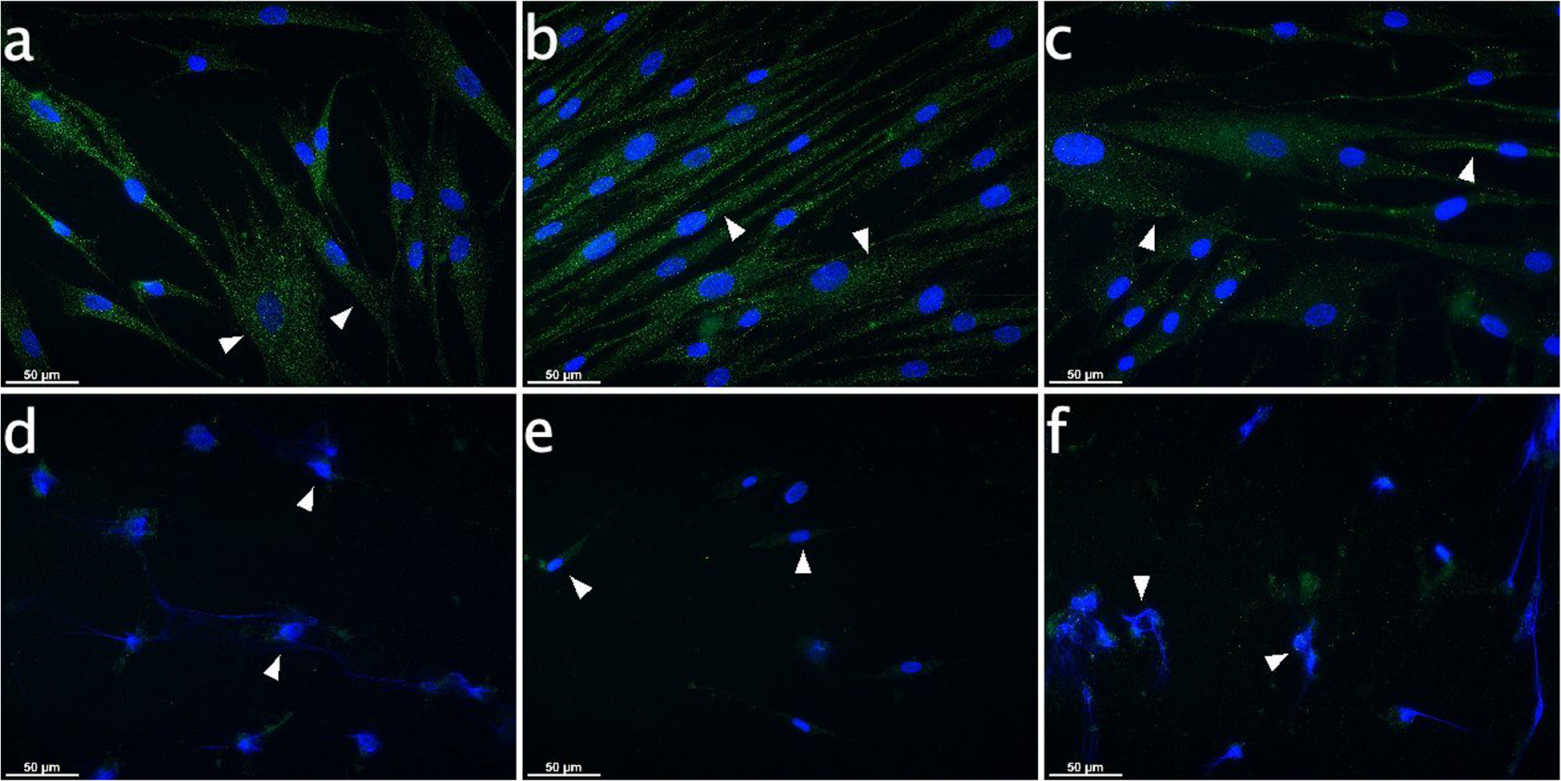
IBA-1 expression in PDL cells subject to CB receptor agonist modulation with or without CII for 10 h. Immunocytochemical analyses for IBA-1 (green) after stimulation for 10 h revealed inherent expression in cultured PDL cells. The IBA-1 signals were distributed equally in the cytoplasm for controls and with endocannabinoid treatment (arrowheads on Fig. **a**, **b**, **c**), exhibiting dotted structures. CII evoked nuclear damage (arrowheads on Fig. **d**, **f**), loss of cells and reversal of IBA-1 signals. However, the more intact nuclei were observed in the CIIspecimens additionally treated with AEA. **a**: Control, **b**: AEA-treated (50 μM), **c**: PEA-treated (50 μM), **d**: 6 h CII, **e**: 6 h CII plus AEA-treated (50 μM), **f**: 6 h CII plus PEA-treated (50 μM). Visualization was performed with Alexa 488 (green) secondary antibody. Nuclei were stained with DAPI (blue). Scale bar is 50 μm

In PDL controls and in specimens exposed to AEA or PEA for 10 h, IBA-1 signals manifested the same dotted structures within the cytoplasm and equal MFI values as seen after 6 h (arrowheads in Fig. 6a-c). In the CII and the CII plus PEA-treated group, IBA-1 signals were completely abrogated and nuclei were damaged (arrowheads in Fig. 6d&f). However, the nuclei were more intact upon CII plus AEA-treatment (arrowheads in Fig. 6e).

**Fig. 7:**
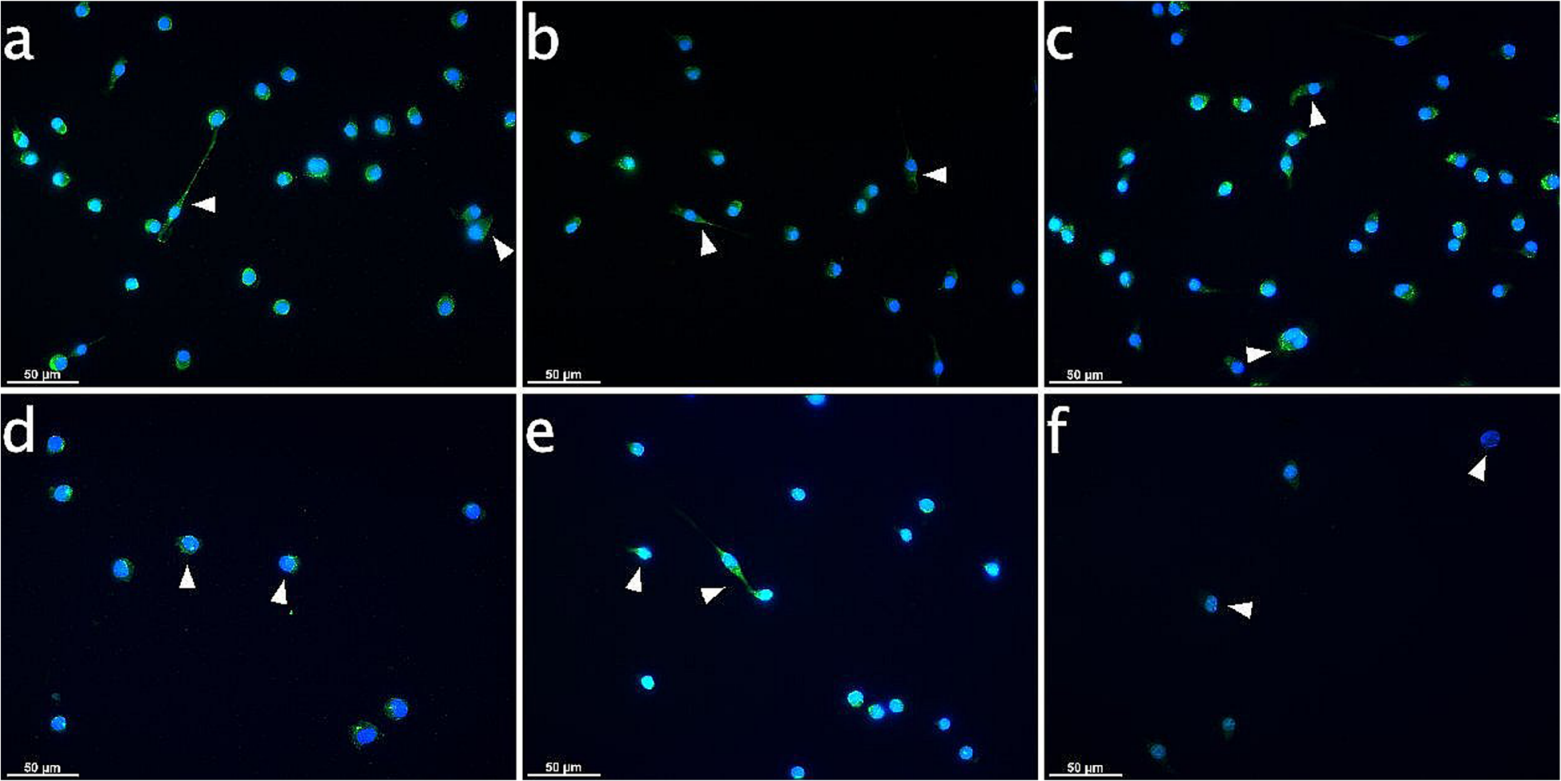
IBA-1 expression in microglia subject to CB receptor agonist modulation with or without CII for 6 h. Immunocytochemical analyses for IBA-1 (green) after stimulation for 6 h in cultured Embryonic stem cell-derived microglia (ESdM) cells. The IBA-1 signals were distributed equally in the cytoplasm for controls and with endocannabinoid treatment (arrowheads on Fig. **a**, **b**, **c**). CII induced loss of cells. IBA-1 MFI values increased in the CII and the CII plus AEA group (arrowheads on Fig. **d**, **e**). In the specimes exposed to CII plus PEA, IBA-1 signals dimished (arrowheads on Fig. **f**). **a**: Control, **b**: AEA-treated (50 μM), **c**: PEA-treated (50 μM), **d**: 6 h CII, **e**: 6 h CII plus AEA-treated (50 μM), **f**: 6 h CII plus PEA-treated (50 μM). Visualization was performed with Alexa 488 (green) secondary antibody. Nuclei were stained with DAPI (blue). Scale bar is 50 μm

In ESdM cells, controls and samples with AEA or PEA for 6 h featured equal signal distribution in the cytoplasm (arrowheads in Fig. 7a-c). MFI values were generally higher than in PDL cells, ranging between 57.9 ± 3.7 and 64.1 ± 2.5.

After CII (Fig. 7d-f), cells were still in shape, but their numbers significantly reduced, with the least cells in the PEA-treated group. For the CII group and the CII plus AEA treated group, IBA-1 signals intensified with MFI values of 68.8 ± 3.7 and 70.4 ± 2.2, but reduced to 21.8 ± 1.3 when stimulated with CII plus PEA.

**Fig. 8:**
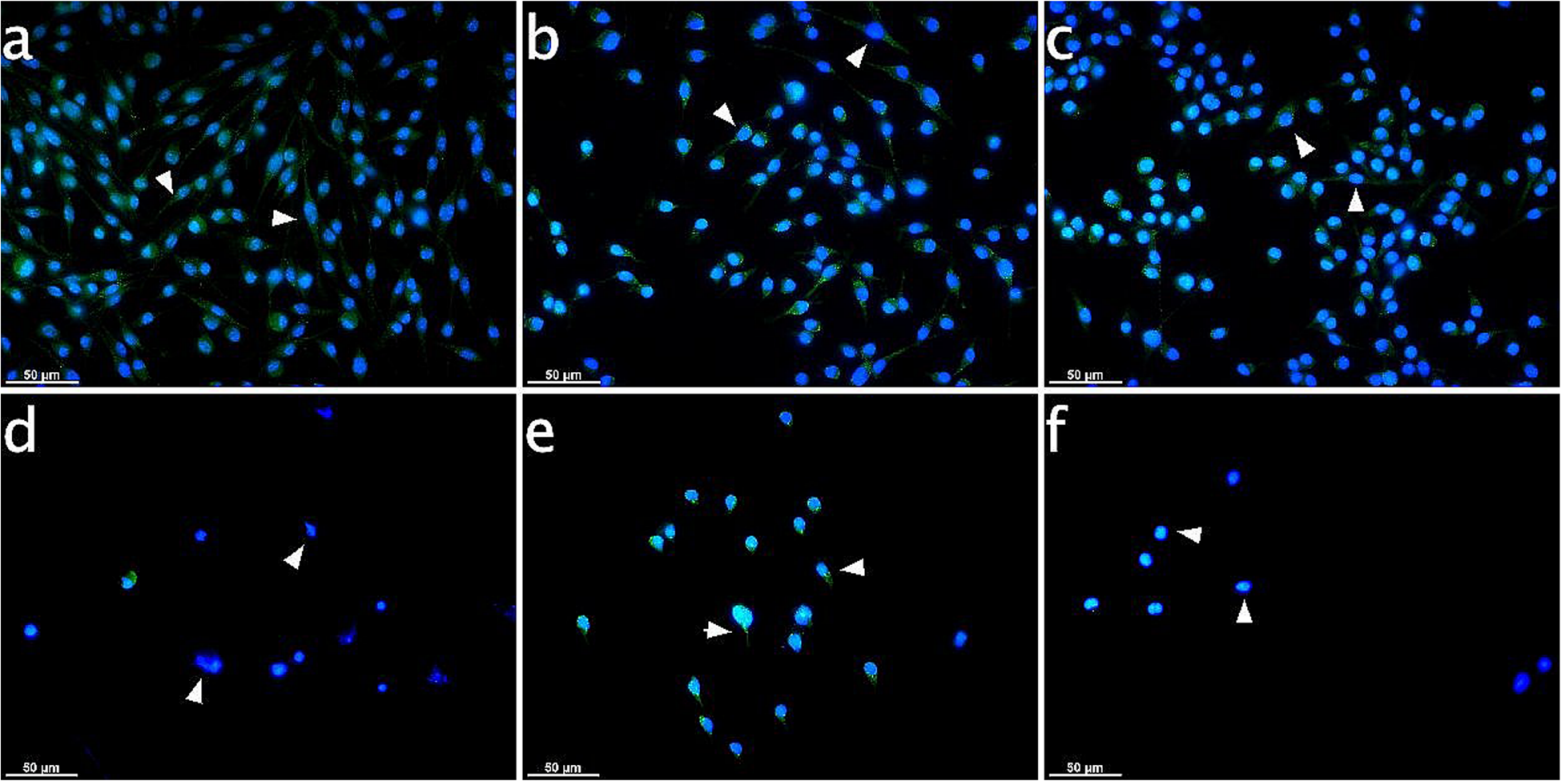
IBA-1 expression in microglia subject to CB receptor agonist modulation with or without CII for 10 h. Immunocytochemical analyses for IBA-1 (green) after 10 h stimulation in cultured Embryonic stem cell-derived microglia (ESdM) cells. The IBA-1 signals were distributed equally in the cytoplasm for controls and with endocannabinoid treatment (arrowheads on Fig. **a**, **b**, **c**). CII effected loss of cells. IBA-1 signal diminished upon CII treatment (arrowheads on Fig. **d** showing cell bodies without fluorescence signal in the cytoplasm). CII plus PEA treatment lead to translocation of IBA-1 signal into the nuclei (arrowheads on Fig. **f**). IBA-1 signal resembled control cells in the CII specimes additionally exposed to AEA (arrowheads on Fig. **e**). **a**: Control, **b**: AEA-treated (50 μM), **c**: PEA-treated (50 μM), **d**: 6 h CII, **e**: 6 h CII plus AEA-treated (50 μM), **f**: 6 h CII plus PEA-treated (50 μM). Visualization was performed with Alexa 488 (green) secondary antibody. Nuclei were stained with DAPI (blue). Scale bar is 50 μm

In ESdM specimens challenged with AEA or PEA for 10 h and in controls, IBA-1 signals remained stable and equally distributed in the cytoplasm like in the 6 h samples (arrowheads in Fig. 8a-c). IBA-1 signals were markedly reduced by CII to an MFI value of 38.4 ± 2.5 and even more in the CII plus PEA-treated group to 19.6 ± 1.7 (arrowheads in Fig. 8d&f). Additionally, CII decreased cell numbers with the least found in PE-treated group. Contrarily, AEA was able to offset these effects and cells maintained IBA-1 signal intensity as seen for controls with an MFI value of 65.3 ± 3.2. Cell numbers also remained unaffected by CII due to the counteracting effects by AEA (Fig. 8e).

**Fig. 9:**
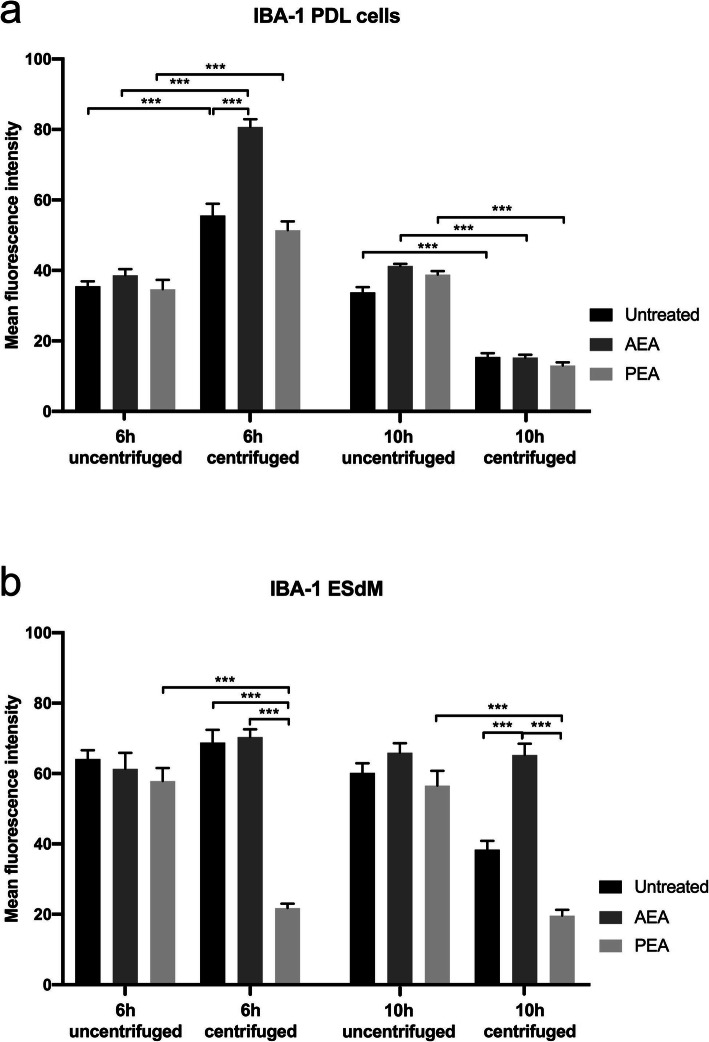
IBA-1 MFI values in PDL cells and microglia subject to CB receptor agonist modulation with or without CII. Quantification of MFI values for IBA-1 staining is illustrated as the mean values following treatments indicated on the x-axis. Values represent the mean ± SEM (*n* = 6). Statistics were performed with one-way ANOVA for the comparison between groups followed by Bonferroni correction. The level for statistical significance was set at *p* < 0.05 (* *p* < 0.05, ** *p* < 0.01, *** *p* < 0.001)

MFI values of IBA-1 in PDL cells and ESdM cells are shown in Fig. 9.

## Discussion

To the best of our knowledge, this study is the first to uncover similarities in the immunomodulatory behavior of PDL cells located in the periodontium and microglia residing in the CNS. Analogous to microglia as innate immune cell type of the CNS, the endocannabinoids AEA and PEA seem to control the regulatory function and performance of PDL cells. In detail, PDL cells inherently expressed IBA-1 both at the transcriptional and protein level. This calcium-binding protein was classically considered to be restricted to microglia cells, but subsequently emerged to be present on fibroblasts, endothelial cells and smooth muscle cells in immune-inflammatory disorders as well [21, 34–36]. CII increased expression of IBA-1 in PDL cells and the staining pattern changed by locating around the nuclei due to a transformation of cell shape from spindle-shaped to roundish structures. These effects seen upon CII with or without PEA was not evident for CII in the presence of AEA, which was able to counteract morphological changes, maintain the physiological cell shape, meaning the morphology of the cell body in an unstimulated and resting status, which is shown in Figs. 5, 6, 7 and 8 in image a), and even increase IBA-1 expression once more. Patterns were quite similar in microglia, where AEA featured reversion of changes in cell structure evoked by CII. Former investigations on microglia activation evidenced a decisive role for IBA-1 in modulating the actin cytoskeleton organization and partaking in membrane ruffling to enable cell migration and phagocytosis as first line of host protection [19]. Other works additionally stated that IBA-1 is highly up-regulated in immune-inflammtory disorders as much as autoimmunology and thus might represent an important molecular target for the modulation of local defense mechanisms [37]. In our study, a cytoskeletal rearrangement and thus change in IBA-1 staining pattern upon CII was not only evident in microglia, but also in PDL cells, indicating a role of this molecule in the activation of periodontal cells. Future investigations should pursue a potential involvement of IBA-1 in PDL cell prearrangement of phagocytosis and migration, representing host protective mechanisms upon immune challenge as found in microglia.

The coordinated inflammatory response in microglia and more precisely in its M1 phenotype is attended by cytokine expression including IL-1ß, TNFα and IL-6, just as it is for PDL cells [4, 5, 38, 39]. This inflammatory capacity of microglia is tightly regulated in order to ensure a return to a surveying state or a regulatory M2 phenotype after acute pathologies are resolved, but the underlying mechanisms are not fully understood to date [40, 41]. The protective and inflammation-resolving attributes discovered for PEA cannot be emphasized by our results, as it aroused pro-inflammatory, cell-destructive and anti-proliferative effects both in PDL cells and microglia.

However, the present findings regarding the function of AEA in PDL cells are conformable to other works showing anti-inflammatory capacities of AEA upon challenge with LPS, TNF-α and IL-1β [13]. Our investigations show that AEA might be a very promising regulator of transient activation upon immune challenge both in PDL cells and in microglia, as it maintains the physiological cell shape and furthermore effectively counteracts up-regulation of inflammatory cytokines evoked by mechanical loading via CII. It was shown that AEA is able to control and restrict immune responses after primary CNS damage over a cannabinoid receptor-mediated pathway, and investigations further evidenced that AEA driven effects in microglia are mainly induced through CB2 receptors [23, 42]. Recent works on PDL cells already found an impact of CB2 on cell adhesion and migration [9]. In the present study, CB receptor regulation in microglia was unaffected by stimulation, indicating a stable expression not being modulated by external influences. Contrarily, characterization of CB1 and CB2 on PDL cells identified a significant induction of both molecules due to CII, which could be potently alleviated by AEA administration analogous to its inflammation-reducing effects. These findings indicate species-related differences regarding CB receptor regulation in immunology, which is supported by other studies propagating an additional, as yet unidentified CB receptor on microglia responsible for inflammatory balance [43].

## Conclusions

Taken together, our findings account for a similar role of PDL cells and microglia in immunological settings each in their corresponding local niche. Here, function of these resident constituents in the development of inflammatory conditions and understanding of their manipulating parameters is critical for the control of periodontal pathophysiology. Host-protective features could be identified for AEA through dampening inflammation and preserving cellular integrity in both cell types, whereas pro-inflammatory and exacerbating effects were aroused by PEA. These observations suggest that the endocannabinoid system might be a promising target in the regulation of periodontal host response. Furthermore, PDL cells exhibit immunological features equaling microglia and hence underline their potential role as non-professional antigen-presenting cells.

## Data Availability

The datasets used and/or analyzed during the current study are available from the corresponding author on reasonable request.

## Abbreviations

PDL cells: Periodontal ligament cells
TNFα: Tumor necrosis factor α
AEA: N-arachidonoylethanolamine
CII: Centrifugation-induced inflammation
CNS: Central nervous system
ESdM: Embryonic stem cell-derived microglia
Iba1: Ionized calcium binding adaptor molecule 1
IL: Interleukin
MFI: Mean fluorescence intensity
PEA: Palmitoylethanolamide
qRT-PCR: Quantitative real-time polymerase chain reaction
ROI: Regions of interest

## References

[1] Konermann A, Beyer M, Deschner J, Allam JP, Novak N, Winter J, Jepsen S, Jäger A (2012). Human periodontal ligament cells facilitate leukocyte recruitment and are influenced in their immunomodulatory function by Th17 cytokine release. Cell Immunol.

[2] Konermann A, Deschner J, Allam JP, Novak N, Winter J, Baader SL, Jepsen S, Jäger A (2012). Antigen-presenting cell marker expression and phagocytotic activity in periodontal ligament cells. J Oral Pathol Med.

[3] Konermann A, Stabenow D, Knolle PA, Held SA, Deschner J, Jäger A (2012). Regulatory role of periodontal ligament fibroblasts for innate immune cell function and differentiation. Innate Immun.

[4] Alhashimi N, Frithiof L, Brudvik P, Bakhiet M (2001). Orthodontic tooth movement and de novo synthesis of proinflammatory cytokines. Am J Orthod Dentofac Orthop.

[5] Page RC (1991). The role of inflammatory mediators in the pathogenesis of periodontal disease. J Periodontal Res.

[6] Li J, Casanova JL, Puel A (2018). Mucocutaneous IL-17 immunity in mice and humans: host defense vs. excessive inflammation. Mucosal Immunol.

[7] Nakajima Y, Furuichi Y, Biswas KK, Hashiguchi T, Kawahara K, Yamaji K, Uchimura T, Izumi Y, Maruyama I (2006). Endocannabinoid, anandamide in gingival tissue regulates the periodontal inflammation through NF-kappaB pathway inhibition. FEBS Lett.

[8] Konermann A, Jäger A, Held SA, Brossart P, Schmöle A (2017). In vivo and in vitro identification of Endocannabinoid signaling in periodontal tissues and their potential role in local pathophysiology. Cell Mol Neurobiol.

[9] Liu C, Qi X, Alhabeil J, Lu H, Zhou Z (2019). Activation of cannabinoid receptors promote periodontal cell adhesion and migration. J Clin Periodontol.

[10] Qian H, Zhao Y, Peng Y, Han C, Li S, Huo N, Ding Y, Duan Y, Xiong L, Sang H (2010). Activation of cannabinoid receptor CB2 regulates osteogenic and osteoclastogenic gene expression in human periodontal ligament cells. J Periodontal Res.

[11] Yan W, Cao Y, Yang H, Han N, Zhu X, Fan Z, Du J, Zhang F (2019). CB1 enhanced the osteo/dentinogenic differentiation ability of periodontal ligament stem cells via p38 MAPK and JNK in an inflammatory environment. Cell Prolif.

[12] Skaper SD, Facci L, Barbierato M, Zusso M, Bruschetta G, Impellizzeri D, Cuzzocrea S, Giusti P (2015). N-Palmitoylethanolamine and Neuroinflammation: a novel therapeutic strategy of resolution. Mol Neurobiol.

[13] Abidi AH, Presley CS, Dabbous M, Tipton DA, Mustafa SM, Moore BM (2018). Anti-inflammatory activity of cannabinoid receptor 2 ligands in primary hPDL fibroblasts. Arch Oral Biol.

[14] Özdemir B, Shi B, Bantleon HP, Moritz A, Rausch-Fan X, Andrukhov O (2014). Endocannabinoids and inflammatory response in periodontal ligament cells. PLoS One.

[15] Pandey R, Mousawy K, Nagarkatti M, Nagarkatti P (2009). Endocannabinoids and immune regulation. Pharmacol Res.

[16] Petrosino S, Cristino L, Karsak M, Gaffal E, Ueda N, Tüting T, Bisogno T, De Filippis D, D'Amico A, Saturnino C, Orlando P, Zimmer A, Iuvone T, Di Marzo V (2010). Protective role of palmitoylethanolamide in contact allergic dermatitis. Allergy..

[17] Vaia M, Petrosino S, De Filippis D, Negro L, Guarino A, Carnuccio R, Di Marzo V, Iuvone T (2016). Palmitoylethanolamide reduces inflammation and itch in a mouse model of contact allergic dermatitis. Eur J Pharmacol.

[18] Kozono S, Matsuyama T, Biwasa KK, Kawahara K, Nakajima Y, Yoshimoto T, Yonamine Y, Kadomatsu H, Tancharoen S, Hashiguchi T, Noguchi K, Maruyama I (2010). Involvement of the endocannabinoid system in periodontal healing. Biochem Biophys Res Commun.

[19] Ohsawa K, Imai Y, Sasaki Y, Kohsaka S (2004). Microglia/macrophage-specific protein Iba1 binds to fimbrin and enhances its actin-bundling activity. J Neurochem.

[20] Ohsawa K, Imai Y, Kanazawa H, Sasaki Y, Kohsaka S (2000). Involvement of Iba1 in membrane ruffling and phagocytosis of macrophages/microglia. J Cell Sci.

[21] Imai Y, Ibata I, Ito D, Ohsawa K, Kohsaka S (1996). A novel gene iba1 in the major histocompatibility complex class III region encoding an EF hand protein expressed in a monocytic lineage. Biochem Biophys Res Commun.

[22] Tam WY, Ma CH (2014). Bipolar/rod-shaped microglia are proliferating microglia with distinct M1/M2 phenotypes. Sci Rep.

[23] Malek N, Popiolek-Barczyk K, Mika J, Przewlocka B, Starowicz K (2015). Anandamide, acting via CB2 receptors, Alleviates LPS-Induced Neuroinflammation in Rat Primary Microglial Cultures. Neural Plast.

[24] Beins E, Ulas T, Ternes S, Neumann H, Schultze JL, Zimmer A (2016). Characterization of inflammatory markers and transcriptome profiles of differentially activated embryonic stem cell-derived microglia. Glia..

[25] Beutner C, Linnartz-Gerlach B, Schmidt SV, Beyer M, Mallmann MR, Staratschek-Jox A, Schultze JL, Neumann H (2013). Unique transcriptome signature of mouse microglia. Glia..

[26] Beutner C, Roy K, Linnartz B, Napoli I, Neumann H (2010). Generation of microglial cells from mouse embryonic stem cells. Nat Protoc.

[27] Redlich M, Asher Roos H, Reichenberg E, Zaks B, Mussig D, Baumert U, Golan I, Palmon A (2004). Expression of tropoelastin in human periodontal ligament fibroblasts after simulation of orthodontic force. Arch Oral Biol.

[28] Redlich M, Palmon A, Zaks B, Geremi E, Rayzman S, Shoshan S (1998). The effect of centrifugal force on the transcription levels of collagen type I and collagenase in cultured canine gingival fibroblasts. Arch Oral Biol.

[29] Davidovitch Z (1991). Tooth movement. Crit Rev Oral Biol Med.

[30] De Petrocellis L, Davis JB, Di Marzo V (2001). Palmitoylethanolamide enhances anandamide stimulation of human vanilloid VR1 receptors. FEBS Lett.

[31] Chiurchiù V, Leuti A, Smoum R, Mechoulam R, Maccarrone M (2018). Bioactive lipids ALIAmides differentially modulate inflammatory responses of distinct subsets of primary human T lymphocytes. FASEB J.

[32] Pfaffl MW (2001). A new mathematical model for relative quantification in real-time RT-PCR. Nucleic Acids Res.

[33] Jensen EC (2013). Quantitative analysis of histological staining and fluorescence using ImageJ. Anat Rec (Hoboken).

[34] Autieri MV, Kelemen SE, Wendt KW (2003). AIF-1 is an actin-polymerizing and Rac1-activating protein that promotes vascular smooth muscle cell migration. Circ Res.

[35] Tian Y, Jain S, Kelemen SE, Autieri MV (2009). AIF-1 expression regulates endothelial cell activation, signal transduction, and vasculogenesis. Am J Physiol Cell Physiol.

[36] Yamamoto A, Ashihara E, Nakagawa Y, Obayashi H, Ohta M, Hara H, Adachi T, Seno T, Kadoya M, Hamaguchi M, Ishino H, Kohno M, Maekawa T, Kawahito Y (2011). Allograft inflammatory factor-1 is overexpressed and induces fibroblast chemotaxis in the skin of sclerodermatous GVHD in a murine model. Immunol Lett.

[37] Kadoya M, Yamamoto A, Hamaguchi M, Obayashi H, Mizushima K, Ohta M, Seno T, Oda R, Fujiwara H, Kohno M, Kawahito Y (2014). Allograft inflammatory factor-1 stimulates chemokine production and induces chemotaxis in human peripheral blood mononuclear cells. Biochem Biophys Res Commun.

[38] Dantzer R (2001). Cytokine-induced sickness behavior: mechanisms and implications. Ann N Y Acad Sci.

[39] Dantzer R, O'Connor JC, Freund GG, Johnson RW, Kelley KW (2008). From inflammation to sickness and depression: when the immune system subjugates the brain. Nat Rev Neurosci.

[40] Fenn AM, Henry CJ, Huang Y, Dugan A, Godbout JP (2012). Lipopolysaccharide-induced interleukin (IL)-4 receptor-α expression and corresponding sensitivity to the M2 promoting effects of IL-4 are impaired in microglia of aged mice. Brain Behav Immun.

[41] Norden DM, Godbout JP (2013). Review: microglia of the aged brain: primed to be activated and resistant to regulation. Neuropathol Appl Neurobiol.

[42] Eljaschewitsch E, Witting A, Mawrin C, Lee T, Schmidt PM, Wolf S, Hoertnagl H, Raine CS, Schneider-Stock R, Nitsch R, Ullrich O (2006). The endocannabinoid anandamide protects neurons during CNS inflammation by induction of MKP-1 in microglial cells. Neuron..

[43] Puffenbarger RA, Boothe AC, Cabral GA (2000). Cannabinoids inhibit LPS-inducible cytokine mRNA expression in rat microglial cells. Glia.

